# Impact of fast-track discharge from cardiothoracic intensive care on family satisfaction

**DOI:** 10.1186/s12871-015-0060-6

**Published:** 2015-05-23

**Authors:** Amr S. Omar, Praveen C. Sivadasan, Mumi Gul, Rula Taha, Alejandro Kohn Tuli, Rajvir Singh

**Affiliations:** 1 Department of Cardiothoracic Surgery/Intensive Care Unit Section, Heart Hospital, Hamad Medical Corporation, PO Box 3050, Doha, Qatar; 2 Department of Critical Care Medicine, Beni Suef University, Beni Suef, Egypt; 3 Department of Medial Research, Hamad Medical Corporation, Doha, Qatar

**Keywords:** Family satisfaction, Intensive care unit, Fast-track discharge

## Abstract

**Background:**

Dissatisfaction with the intensive care unit may threaten medical care. Clarifying treatment preferences can be useful in these settings, where physician direction may influence decision making and therefore medical treatment. This study aimed to evaluate whether fast-track discharge from intensive care units affects the satisfaction of family members.

**Methods:**

We used a single-center non-randomized trial, with all eligible family members involved. To evaluate family satisfaction, we used the Society of Critical Care Family Needs Assessment questionnaire (SCCMFNAQ). We hypothesized that those discharged within 24 h of intensive care unit admission and their families would have higher levels of satisfaction. Patients were scored using the therapeutic interventions scoring system (TISS) and additive EuroSCORE.

**Results:**

Two-hundred fifty-five family members were enrolled. The mean patient age was 53 years, and 92 % were male. The median satisfaction level among family members was 17.9 (range 14–31). Patients were divided into two groups, one receiving fast-track discharge (116 patients), and one whose members stayed longer (139 patients). The overall satisfaction was affected significantly by quality of the delivered care and dissatisfaction increased by lack of comfort in hospital settings, including the waiting room. No significant differences were seen between the two groups for overall satisfaction (*p* = 0.546) and individual components of the questionnaire. Higher satisfaction was linked to higher levels of education among family members (*p* = 0.045) and information being relayed by a senior physician *p* = 0.03 (two-tailed test).

**Conclusions:**

Fast-track discharge from intensive care did not influence family satisfaction as hypothesized. Satisfaction relied on family members’ level of education and the level of seniority of the physician relaying information.

## Background

One of the primary initiatives to promote care for acutely ill patients is to create an outline of the patient’s treatment preferences [1]. This is particularly relevant in the intensive care unit (ICU) because the severity of a patient’s condition may limit his/her decision-making capacity [2]. Advanced directives are often neither implemented by physicians nor initiated by patients, leading to failure of clinical decision guidance [3]. The ability of patients and their families to understand treatment directions can be further complicated by physicians’ attitudes and practices, which are usually related to the achieved satisfaction level [4]. Numerous studies from the United States and Europe have documented the needs of family members of critically ill patients because they are frequently involved in psychological crises [5, 6]. Providing adequate care for patients and families has emerged as a priority for ICU physicians and nurses [5, 6].

The sensation of being cared for, along with a sense of security in families who are deeply involved in a life crisis, are related to psychological assurance from ICU physicians and nurses. Information that is provided by health care professionals needs to be clear and appropriate to engage family members in making decisions about patients. Some studies on family satisfaction in ICUs [5, 7] have suggested that information giving is one of the most effective means of communication. This should be considered a cornerstone in successful interventions for crises with families of ICU patients. Family satisfaction is improved by clear information, but provision of reassurance and professional closeness to the patient is as important as the need for communication [5–9].

Few studies have been carried out in the Middle East to measure satisfaction of family members of ICU patients [10, 11] and none have been carried out in Qatar. Qatar is a multinational community, with more than 50 nationalities receiving treatment at Hamad Medical Corporation. Therefore, communication between health care professionals and the patients’ families can be complicated. Arab people are highly dependent on their family members to make surrogate decisions related to treatment options. Keeping family members informed regarding the patient’s condition and talking to the family member in confidence are important [12].

Klingele et al. defined “fast-track discharge” from the ICU as discharge within 24 h of initial admission [10]. We hypothesize that fast-track discharge from the ICU has a positive effect on overall family satisfaction.

The study aimed to evaluate the satisfaction of family members of patients in a Qatari ICU, and to assess determinants of satisfaction, particularly the relationship with fast-track discharge from the ICU. We also aimed to determine if family satisfaction could be improved through an effective assessment and communication plan.

## Methods

This was a prospective, single-center survey with purposive sampling of patients’ family members. The survey was conducted in a 12-bed, post-cardiac-surgery ICU of Heart Hospital, Hamad Medical Corporation. This ICU admits approximately 300 patients annually with an average daily turnover of two patients who have an average age of 40 years. Staff included six consultants and 15 specialists. We included family members who were older than 18 years, adult patients older than 16 years, and family members and patients who were available and agreed to participate in the study. We excluded families who could not be traced or refused to participate, and in cases of poor understanding of the questionnaires.

There were 321 patients whose family members were eligible, of whom 15 family members refused to participate, and a further 51 could not be traced. The remaining 255 patients were included in the study. The study was conducted from September 2012 to October 2013 after approval by the ethics committee (Hamad Medical Corporation-Institution review board: reference number 13244/13), which waived the requirement for informed consent. Questionnaires were provided to family members after the daily round, which included the list of problems and plan of management. A physician met the family members of each patient in the ICU, and asked them to complete the form.

The following information was recorded for each patient: age, sex, marital status, diagnosis, length of stay in the ICU, European System for Cardiac Operative Risk Evaluation (EuroSCORE) to assess the effect of variations in patients’ risk profiles [13], the Therapeutic Intervention Scoring System (TISS) score on the day of the interview [14], use of mechanical ventilation, number of patients in the ICU at the time of interview, and the nurse-to-patient ratio. Demographic information for each family member included the following: age, sex, nationality, relationship with the patient, occupation, level of education, commuting time to the ICU per day, and whether information had been provided by a senior or junior ICU physician. Family members were defined as all individuals (relatives or friends) who visited the patient in the ICU, regardless of their relationship to the patient [5].

Satisfactory understanding of the diagnosis was defined as having knowledge of the diagnosis before admission. Comprehension of the treatment was defined as knowledge of the most important treatments performed on admission [15]. Finally, satisfactory comprehension of prognosis was considered as knowing whether the patient was expected to survive [16].

We used the Society of Critical Care Medicine’s family needs assessment questionnaire (SCCMFNAQ), validated by Johnson et al. to assess family satisfaction [5]. This instrument consists of 14 items, each rated on a four-point self-report Likert scale, ranging from 1 (extreme satisfaction) to 4 (extreme dissatisfaction). The final satisfaction score is calculated as the sum of the scores of all 14 items. Therefore, the smallest possible score is 14, indicating total satisfaction and the highest possible score is 56, indicating extreme dissatisfaction. Because of the multicultural atmosphere, Arabic, English, Hindi, and Urdu versions of the SCCMFNAQ were made available. The questionnaire was slightly reworded based on family members’ suggestions. A physician was available in case of questions during the discussion.

Immediately after the daily visit to the ICU, a researcher approached every family representative and provided verbal information on the purpose and procedures of the study. The visitors were asked to anonymously complete the SCCMFNAQ. The questionnaire was provided to family members 2 or 3 days postoperatively. The form was recovered immediately after completion and family members were not allowed to take the questionnaires home. The effect of the information provided by nurses and paramedics was not evaluated. We compared the individual components of the SCCMFNAQ score with the mean SCCMFNAQ score (total SCCMFNAQ score divided by 14). A pilot group of 20 family members was used to test the validity and reliability of the questionnaires, and this was not included in the final sample.

The TISS is a scoring system denoting the intensity of interventions carried out on the patient. The total maximum score is 78. Interventions are allocated scores ranging from 1–4, depending on the severity. The severity of intervention is assigned to one of the four classes depending on the severity [17].

Normally distributed continuous variables are reported as mean ± SD and non-normally distributed continuous variables are shown as median and range. Categorical variables are reported as frequency and percentage. Normally and non-normally distributed continuous variables were compared using the Student’s *t*-test and Mann–Whitney *U* test, respectively. Categorical variables were compared using the chi-squared test. A two-sided *p* value < 0.05 was considered statistically significant. The reliability of the translated version of the SCCMFNAQ was addressed using Cronbach’s alpha coefficient. A coefficient of ≥ 0.70 suggests that the items within the scale measure the same construct, which strengthens the construct validity [18]. All statistical analyses were carried out using SPSS for Mac 22.0 (SPSS, USA).

## Results

Completed questionnaires were collected from 255 family members of patients and included in this study. The patients were predominantly male. Detailed demographic and surgical characteristics are shown in Table 1. Intraoperative and ICU details, including the severity of illness scoring, are shown in Table 2. Demographic characteristics of relatives and the details of administration of SCCMFNAQ scores are shown in Table 3.

**Table 1:** Demographic and surgical characteristics of patients in the ICU

Characteristic		Mean ± SD[median(min-max)] N (%)
Sex	Male	235 (92.2)
	Female	20 (7.8)
Age (years)	52.85 ± 11.55 [54 (19–81)]	
	Less than 20	1 (0.3)
	21–30	12 (4.7)
	31–40	23 (9.0)
	41–50	62 (24.3))
	51–60	96 (37.6)
	61–70	50 (19.6)
	71–80	11 (4.3)
	More than 80	1 (0.3)
Surgical procedure	Isolated CABG	180 (70.6)
	CABG + valve	4 (1.6)
	CABG + valve + other	2 (0.8)
	CABG + others	3 (1.2)
	isolated valve	40 (15.7)
	Valve + aortic/ablation	14 (5.5)
	Others	12 (4.7)
Baseline EF %		49.74 ± 10.32 [51 (20–65)]
Pre op cardiogenic shock		8 (3.1 %)
Pre op IABP		9 (3.5 %)
Pre op mechanical ventilation		6 (2.4 %)
Baseline creatinine (mmol/l)		89.62 ± 33.963 [84 (38–411)
Additive Euroscore		3.59 ± 3.387 [3 (0–20)]
operative urgency (elective: urgent: emergency: salvage)		158:81:10: 6

*CABG* coronary artery bypass graft, *EF* ejection fraction, *IABP* intra-aortic balloon pump

**Table 2:** Intra-operative and ICU data

Characteristic		Mean ± SD[median (min-max)] N (%)
CPB time (minutes)		114.42 ± 47.03 [103 (30–317)]
ACC time (minutes)		72.52 ± 38.86 [63 (11–251)]
IABP		16 (6.3 %)
Re-exploration		22 (7.9 %)
ICU blood products given (NO OF UNITS)	PRBC	1.96 ± 2.817 [0 (0–18)]
	FFP	.84 ± 2.019 [0 (0–12)]
	Platelets	2.15 ± 5.261 [0 (0–36)]
ICU total blood loss (ml)		965.3 ± 786.48 [765 (50–6600)]
TISS Score		53.19 ± 11.12 [49 (36–88)]
Renal Complications	AKI	70 (27)
	New dialysis	2 (0.8)
Pulmonary complications		8 (3.1)
Infectious complications		10 (4)

*CPB* cardiopulmonary bypass, *ACC* aortic cross clamp, *IABP* intra-aortic balloon pump, *PRBCs* packed red blood cells, *FFP* fresh frozen plasma, *AKI* acute kidney injury

**Table 3:** Family members’ data

Characteristic		Mean ± SD [median (min-max)] N (%)
Age		45.60 ± 9.263 [45 (18–68)]
Sex	Male	124 (48.6)
	Female	131 (51.4)
Educational level	None	5 (2.0)
	elementary	119 (46.7)
	high school	76 (29.8)
	Higher	55 (21.6)
SCCMFNAQ administration	self	215 (84.3)
	Staff	40 (15.7)
Information given to family by	consultant	39 (15.3)
	Specialist	216 (84.6)

**Fig. 1:**
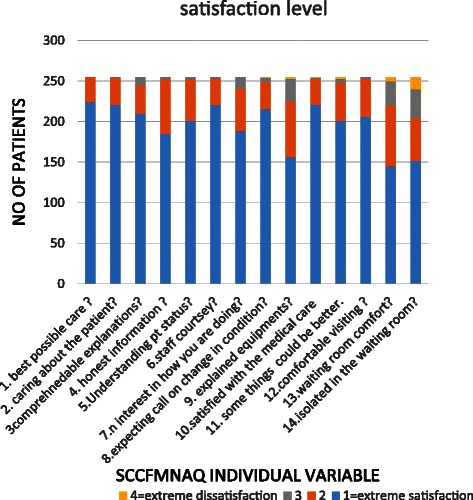
Satisfaction level of patients’ families

**Fig. 2:**
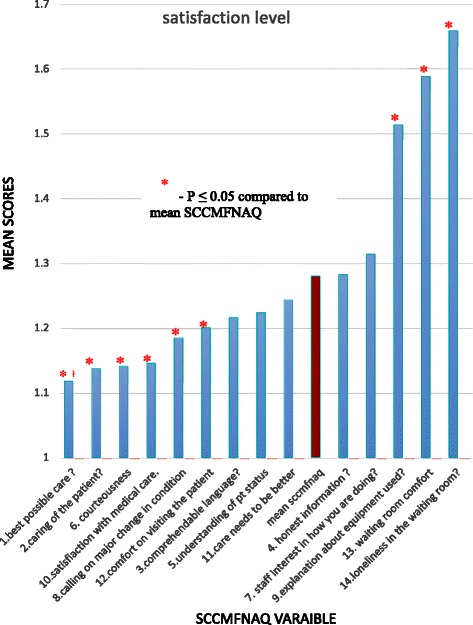
Individual satisfaction mean scores vs mean SCCMFNAQ score

Figure 1 shows the mean satisfaction scores of SCCMFNAQ individual variables in family members. Most of the relatives expressed extreme satisfaction for most of the variables in the questionnaire. Compared with the mean SCCMFNAQ score, family members were significantly more satisfied about delivery of the best possible care, the care of the patient by hospital staff, staff courtesy, and the expectation that they would call if there was a change in the patient’s condition. However, they were significantly more dissatisfied with the explanation of the equipment used, the comfort of the waiting room, and feeling isolated while waiting (Fig. 2).

The patients were divided into subgroups: group 1 stayed one day or less in the ICU (116 patients) and group 2 stayed longer than 1 day (139 patients). There was no significant correlation between the length of stay and family satisfaction (Table 4). There were also no significant differences in satisfaction of the individual variables of the SCCMFNAQ score between the two groups.

**Table 4:** Individual parameters of the SCCMFNAQ according to the length of stay

	Group I (116 patients) (mean ± SD)	Group II (139 patients) (mean ± SD)	Significance (2-tailed)
1) Best possible care being given?	1.10 ± 0.31	1.13 ± 0.34	.520
2) Hospital personnel care about the patient?	1.10 ± 0.31	1.17 ± 0.39	.339
3) Understandable explanations	1.16 ± 0.46	1.26 ± 0.53	.175
4) Honest information?	1.25 ± 0.45	1.31 ± 0.49	.611
5) Understand what is happening to the patient?	1.23 ± 0.42	1.22 ± 0.46	.152
6) Staff courtesy	1.12 ± 0.33	1.16 ± 0.41	.417
7) Staff interest in how you are doing?	1.33 ± 0.60	1.30 ± 0.55	.613
8) Expect to call on change in the patient’s condition?	1.16 ± 0.42	1.20 ± 0.50	.814
9) Explained the equipment being used?	1.55 ± 0.75	1.48 ± 0.70	.834
10) Satisfied with the medical care?	1.15 ± 0.42	1.14 ± 0.37	.557
11) Some things about medical care could be better.	1.24 ± 0.52	1.24 ± 0.52	.994
12) Comfortable visiting the patient?	1.18 ± 0.41	1.22 ± 0.43	.739
13) Waiting room comfort?	1.63 ± 0.81	1.55 ± 0.74	.368
14) Alone and isolated in the waiting room?	1.58 ± 0.81	1.73 ± 1.01	.158
SCCMFNAQ SCORE (MEAN ± SD)	17.86 ± 2.816	18.11 ± 3.7	0.546

Further analysis showed that certain patients’ characteristics, such as prolonged ventilation, the need for further operations, being confused, renal failure, having an intra-aortic balloon pump, and infectious complications were associated with a prolonged ICU stay (Table 5). Re-admission to the ICU was the same in both groups (three times in each group) Similarly, a low baseline ejection fraction, increased bleeding and transfusion rates, a high TISS score, a high pre-operative EuroSCORE, and prolonged bypass were associated with a prolonged stay (Table 6), but none of these parameters affected the satisfaction levels (Table 7).

**Table 5:** Categorical variables according to the length of stay

Variable		Group I (116 patients) (mean ± SD)	Group II (139 patients) (mean ± SD)	*P* value
Sex- male		101 (87.8 %)	133 (95.7 %)	.021
Pre op mechanical ventilation		1 (0.9 %)	5 (3.6 %)	0.152
Cardiogenic shock		1 (0.9 %)	7 (5.1 %)	0.057
Pre op IABP		2 (1.7)	7 (5.1 %)	0.154
Operative urgency	elective	77 (67.0 %)	81 (58.3 %)	0.145
	urgent	35 (30.4 %)	45 (32.4 %)	
	emergency	2 (1.7 %)	8 (5.8 %)	
	salvage	1 (0.9 %)	5 (3.6 %)	
surgery	CABG	88 (76.5 %)	91 (65.5 %)	0.092
	Valve	19 (16.5 %)	21 (15.1 %)	
	CABG+ valve	0 (0 %)	4 (2.9 %)	
	valve + other	4 (3.5 %)	10 (7.2 %)	
	CABG+ valve + other	0 (0 %)	2 (1.4 %)	
	others	4 (3.5 %)	8 (5.8 %)	
	CABG + OTHER	0 (0 %)	3 (2.2 %)	
Reoperation		0 (0 %)	22 (15.8 %)	0.002
Pulmonary complications		0 (0 %)	8 (5.8 %)	0.145
Renal failure		0 (0 %)	9 (6.4 %)	0.021
Confusional state		0 (0 %)	6 (4.3 %)	0.024
Infective complications		0 (0 %)	10 (7.2 %)	0.03
IABP use		2 (1.7 %)	14 (10.1 %)	0.023
Readmission to ICU		3 (2.6 %)	3 (2.1 %)	
LOV	on table extubation	4 (3.7 %)	1 (0.7 %)	0.000
	less than 12 h	98 (90.7 %)	84 (61.8 %)	
	12–24 HR	65.6 %)	30 (22.1 %)	
	≥24 HR	0 (0 %)	21 (15.4 %)	

*CABG* coronary artery bypass graft, *IABP* intra-aortic balloon pump, *LOV* length of ventilation

**Table 6:** Continuous variables according to the length of stay

	Group I (116 patients) (mean ± SD)	Group II (139 patients) (mean ± SD)	*P* value
Patient Age	53.50 ± 11.243	52.35 ± 11.853	.592
EF %	50.81 ± 8.845	48.80 ± 11.40	.007
Creatinine	85.10 ± 18.492	93.24 ± 42.317	.050
ICU Platelets	0.98 ± 2.489	3.14 ± 6.608	.000
Total platelet	1.92 ± 3.320	5.67 ± 8.679	.000
ICU FFP	0.19 ± 0.837	1.39 ± 2.504	.000
Total FFP	0.37 ± 1.096	2.10 ± 3.508	.000
ICU PRBC	0.70 ± 1.003	2.91 ± 3.334	.000
Total PRBC	1.30 ± .391	4.06 ± 4.455	.000
CPB (minutes)	99.83 ± 38.365	126.32 ± 49.975	0.014
Additive EuroScore	2.88 ± 2.193	4.19 ± 4.012	.000
Total blood loss	688.17 ± 300.975	1194.85 ± 972.021	.000
TISS	47.05 ± 5.788	58.36 ± 11.952	.000

*EF* ejection fraction, *FFP* fresh frozen plasma, *PRBCs* packed red blood cells, *CPB* cardiopulmonary bypass, *TISS* Therapeutic Intervention Scoring System

**Table 7:** Pearson correlations and significance of the SCCMFNAQ with individual variables

	Pearson correlation	Sig. (2-tailed)
Current age	−.119	.058
EF %	.005	.934
Creatinine	−.036	.577
ICU platelets	−.071	.258
Total platelet	−.062	.328
ICU FFP	−.060	.336
Total FFP	−.032	.613
CPB time	.014	.818
ACC	.008	.908
Blood loss at 12 h	.003	.966
Blood loss at 24 h	.006	.924
Total blood loss	−.025	.690
Additive Euroscore	−.059	.371
TISS	−.084	.179
Age (family member)	−.024	.707
Sex (family member)	−.043	.491
Occupation	−.080	.204
Nationality	−.041	.44
Level of education	.30	.045
Information given by senior physician	.4	0.03

*EF* ejection fraction, *FFP* fresh frozen plasma, *PRBCs* packed red blood cells, *CPB* cardiopulmonary bypass, *ACC* aortic cross clamp, *TISS* Therapeutic Intervention Scoring System

## Discussion

To the best of our knowledge, this is the first Middle Eastern study to evaluate the effect of information provided to family members of ICU patients. The main findings of our study were that family members tended to be dissatisfied with their own comfort levels while visiting, especially the comfort of the waiting room and the fact that they felt isolated there. Satisfaction was increased by feeling that the patient was obtaining the best possible care and that hospital personnel were taking care of them, staff courtesy, the expectation that they would be called if there was a change in the patient’s condition, and comfort when they visited the patient (the first six items in the questionnaires, Fig. 2). Fast-track discharge did not affect the level of satisfaction (Table 4). Satisfaction measured by questionnaires was significantly related to the level of education and whether information was provided by a senior or junior physician.

Most previous studies on family satisfaction were multicenter or limited to medical or mixed ICUs. Our study was performed in a cardiothoracic surgical ICU setting, which makes it unique. The unpredictability of complex open heart surgery might have an effect on family satisfaction.

Satisfaction in critically ill patients is a complex emotion, and is affected by the interaction between perception and expectations [19]. Satisfying patients’ family members is also a fundamental part of the ICU physicians’ responsibilities because family members need clarification about the patient’s treatment and interventional preferences [19]. We hypothesized that fast-track discharge has a positive effect on family satisfaction which was not substantiated in our study.

Family needs have been evaluated in many studies in Western countries [15, 16, 20] and in a few studies in Arab countries [21]. The median score of the SCCMFNAQ was 17 in our study, highlighting higher satisfaction than in previous studies. We found that the satisfaction was significantly increased for questions 1, 2, 6, 10, 8, and 12. Similarly, Roberti and Fitzpatrick [22] found that the given variables significantly affected satisfaction. However, Neves et al. [23] found that communication and satisfaction with medical care positively influenced overall satisfaction.

Factors significantly affecting dissatisfaction in our study were those in questions 9, 13, and 14, which is similar to the findings of Damghi et al. [21]. Discomfort related to the waiting area was found to affect satisfaction in other studies where relatives experienced “the emotional hell of waiting” [24–26]. In our hospital, the waiting area is a long way from the cardiothoracic ICU. This area is also small and shared with those visiting the coronary care, catheterization, and high-dependency units. Because of the high number of day-case procedures, waiting is often busy during the day.

### Demographic and cultural variables

Qatar’s population is multinational, and was estimated to be approximately 1.6 million in June 2010. Major ethnic groups include Arabs, those from the Indian subcontinent, and also the Philippines. Major religions include Islam, Christianity, Hinduism, and Buddhism. The majority of the Qatari population is Muslim, a tradition in which family is the basic subunit, with close relations between family members. Islamic traditions encourage supporting and visiting those who are sick. Muslims believe that critical illness and death are God’s will, and that family prayers can help overcome these life crises [21]. No single authority exists in the culture of the Indian sub-continent, and diverse opinions and actions are observed. The ideal of longevity is a motive for families to demand life-supporting measures. Many Eastern religions support reverence for the deceased and accept death as a natural event. Compassion is a biphasic act between families and patients in these cultures [27]. Cultural and religious norms suggest lower demands by families of patients in the ICU because acute illness is perceived as predestined and not the caregiver’s fault [28].

**Fig. 3:**
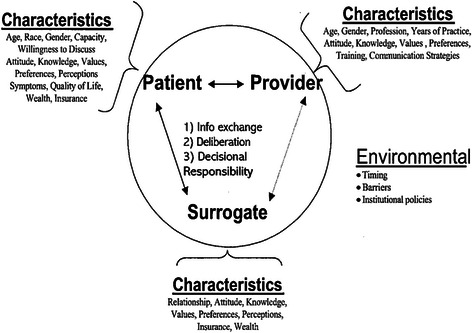
Conceptual framework of a patient–physician interaction. Adopted from Heyland et al. [29]

In the current study, clinical variables that significantly increased the ICU stay were the need for a further operation, renal failure, infective complications, intra-aortic balloon pump use, and the duration of mechanical ventilation (Table 5). Other factors that differed between the two groups of patients included lower ejection fraction, and higher total blood loss, transfusion of blood products, bypass time, additive EuroSCORE, and TISS in more than 1 day stay in ICU group. There was no mortality in our study patients. We hypothesized that satisfaction is affected by the length of ICU stay because of the comorbid condition of the patients. However, our results failed to substantiate this hypothesis. Our finding is in agreement with previous studies. Hunziker et al. [25] found that the length of ventilation, but not the total length of stay, affected satisfaction. Heyland et al. [29] also observed that satisfaction scores were better in patients who were ventilated for more than 48 h, the authors had further provided a conceptual framework of a patient–physician interaction (Fig. 3). Neither the length of ventilation nor the total length of stay affected satisfaction in another study [21]. Families of patients who died while in the ICU were more satisfied than the families of survivors. The centered aspect of care is highly rated, and those who die also stay longer [30]. Azoulay et al. [31] found no relation between satisfaction and severity of illness, the length of hospital stay, or mortality. Another study [32] showed that written admission and discharge criteria were associated with dissatisfaction among family members, which is not consistent with our results.

### Participants’ characteristics

In our study, the SCCMFNAQ was nor correlated with individual variables, but increased satisfaction was significantly related to a higher level of education and information being provided by a senior physician (Table 7). Sundararajan et al. [33] reported that dissatisfaction with care is more frequently found in non-graduates. However, another study showed that better education may be related to a lower level of satisfaction [31]. Lower levels of education in family members were also associated with increased family satisfaction in other studies [23, 34]. Our finding on education level is similar to that by Verhaeghe et al. [35], who showed that a higher education level was associated with increased satisfaction in multivariate analysis.

In a multicenter study by Azoulay et al. [31], the effect of whether information was delivered by a senior or junior physician was investigated. Family members were found to prioritize receiving information from one person, regardless of whether they were senior or junior, rather than from multiple practitioners, because they found it resulted in less contradictory information. Other investigators found that family satisfaction tended to increase in those with lower education levels when information was provided by a senior physician. Developing structured communication programs by health care professionals could be an option to improve satisfaction [34].

We found that information being delivered by a senior physician significantly increased satisfaction (Table 7). This finding might be because Arabs and Asians tend to trust and respect older physicians more than younger physicians. This is consistent with a Moroccan study where families’ preferences were for a senior physician to provide information, and this tended to increase satisfaction [21]. A multicenter French study concluded that junior physicians might lack the time to effectively provide information [29]. Families want daily information on ongoing care, treatment, and interventions provided. Additionally, communication skills and awareness are required, which develop as physicians mature. Junior doctors may be perceived as less proficient. Finally, families’ perceptions might be more negative when information is provided by junior doctors [36].

### Strengths and limitations

A strength of our study is that, to the best of our knowledge, this was the first study to address family satisfaction in a multicultural community, with variable languages and religions. Additionally, this was the first study to use questionnaires in four languages. In contrast to our hypothesis, we found that family satisfaction was not affected by fast-track discharge in the ICU, but rather by high quality care and improved hospital facilities. This study was limited by being performed at a single center, and restricted to a cardiothoracic ICU.

## Conclusions

Fast-track discharge from ICU does not affect family satisfaction. Family satisfaction is dependent on the family member’s level of education and the seniority level of the physician providing information.

### Recommendations and future directions

1. Senior physicians should relay information to families to maximize satisfaction levels.
2. The family member’s level of education should be considered in assessing their satisfaction.
3. Improving the quality of care and the comfort of waiting areas is important.
4. Hospitals should consider establishing a structured communication program for family members of ICU patients, including education of junior physicians.
5. We recommend that management recognizes the importance of care environments in assessing patient and family satisfaction with services.

## Abbreviations

ACC: Aortic cross clamp
AKI: Acute kidney injury
CABG: Coronary artery bypass graft
CPB: Cardiopulmonary bypass
SCCMFNAQ: Society of Critical Care Medicine Family Needs Assessment questionnaire
TISS: Therapeutic Intervention Scoring System
